# Comparative Assessment and High-Throughput Drug-Combination Profiling of TEAD-Palmitoylation Inhibitors in Hippo Pathway Deficient Mesothelioma

**DOI:** 10.3390/ph16121635

**Published:** 2023-11-21

**Authors:** Lale Evsen, Patrick J. Morris, Craig J. Thomas, Michele Ceribelli

**Affiliations:** Division of Preclinical Innovation, National Center for Advancing Translational Sciences (NCATS), National Institutes of Health (NIH), Rockville, MD 20850, USA

**Keywords:** TEAD, TEAD-palmitoylation, TEAD inhibitors, high-throughput drug screening, drug combination, Hippo signaling, malignant pleural mesothelioma, YAP

## Abstract

The hippo signaling pathway is a central tumor suppressor cascade frequently inactivated in selected human cancers, leading to the aberrant activation of TEAD transcription factors. Whereas several TEAD auto-palmitoylation inhibitors are currently in development, a comprehensive assessment of this novel drug-modality is missing. Here, we report a comparative analysis among six TEADi(s) using cell-based and biochemical assays in Hippo pathway deficient mesothelioma. Our analysis revealed varying potency and selectivity across TEADi, also highlighting their limited efficacy. To overcome this limitation, we performed an unbiased, quantitative high-throughput drug screening by combining the TEADi VT-103 with a library of approximately 3000 oncology-focused drugs. By exploiting this library’s mechanistic redundancy, we identified several drug-classes robustly synergized with TEADi. These included glucocorticoid-receptor (GR) agonists, Mek1/2 inhibitors, mTOR inhibitors, and PI3K inhibitors, among others. Altogether, we report a coherent single-agent dataset informing on potency and selectivity of TEAD-palmitoylation inhibitors as single-agents. We also describe a rational pipeline enabling the systematic identification of TEAD druggable co-dependencies. This data should support the pre-clinical development of drug combination strategies for the treatment of Hippo-deficient mesothelioma, and more broadly, for other cancers dependent on the oncogenic activity of YAP/TEAD.

## 1. Introduction

The Hippo signaling pathway is a highly conserved signal transduction cascade acting as a central regulator of organ size control and tissue homeostasis during development [1]. Hippo integrates a diverse set of extracellular signals, including cell-to-cell contact, cell polarity, mechanical cues, and surface receptors engagement into a highly conserved protein kinase cascade ultimately inhibiting cell proliferation [2,3,4]. Downstream of these events, the MST1 and MST2 kinases (Mammalian sterile 20-like 1/2) phosphorylate and activate the LATS1 and LATS2 (Large tumor suppressor homolog 1/2). Once activated, LATS1/2 kinases phosphorylate the transcriptional coactivators YAP/TAZ (“Yes-associated protein” and “Transcriptional co-activator with PDZ binding motif”), inhibiting their function via 14-3-3 dependent cytoplasmic sequestration and/or phosphorylation-induced proteasomal degradation, ultimately restricting cell proliferation [4,5].

Consistent with its central tumor-suppressor role, somatic alterations of the core Hippo pathway genes have been observed in various cancer types, including malignant pleural mesothelioma, cervical squamous cell carcinoma, meningioma, head and neck squamous cell carcinoma, uveal melanoma, and cholangiocarcinoma, among others [6,7,8]. In about 30–40% of malignant pleural mesothelioma cases, for example, the *NF2* (Neurofibromatosis type 2) tumor suppressor gene is somatically inactivated, leading to defective recruitment of LATS1/2 kinases to the plasma membrane and impaired Hippo signaling [9,10]. Mechanistically, defects within this tumor-suppressor pathway cause the aberrant activation of transcriptional coactivators YAP/TAZ, which interact with TEADs transcription factors (TEA domain transcription factors) to promote cell proliferation and oncogenesis. Notably, a regulatory, auto-palmitoylation pocket on TEAD family members has been identified [11,12,13,14,15,16], and several TEAD auto-palmitoylation inhibitors, TEADi(s), are currently in preclinical and clinical development for the treatment of Hippo-deficient cancers [17,18,19,20,21,22,23,24,25,26,27,28,29]. Whereas these collective efforts have provided proof of principle validation of TEAD as a bona-fide druggable target for oncology indications, a comparative assessment among different TEAD-palmitoylation inhibitors is currently lacking. Moreover, whereas some of these early findings offered preliminary insights into the combinatorial potential of TEAD inhibition [18,27,30], particularly in the context of EGFR/RAS/MAPK signaling pathway and resistance to the corresponding targeted agents, an unbiased and comprehensive assessment of TEADi drug-interaction landscape is currently lacking.

Here, we report a compare/contrast analysis among six TEAD-palmitoylation inhibitors, including both covalent and non-covalent small molecules, using Hippo pathway deficient mesothelioma as a model system for YAP/TEAD dependent cancers. We also describe an unbiased, drug-combination screening pipeline that enabled the systematic identification of pairwise, druggable co-dependencies centered around TEAD inhibition in Hippo-deficient pleural mesothelioma.

## 2. Results

### 2.1. Comparative Assessment of TEAD-Palmitoylation Inhibitors in Malignant Pleural Mesothelioma

We selected six TEAD auto-palmitoylation inhibitors based on the published literature, structural diversity, and commercial availability at the time this study was started. These included the reversible small molecules MGH-CP1 [20,27], VT-103 [22], VT-107 [22], and MSC-4106 [31], together with the covalent inhibitors MYF-01-37 [19,23] and K-975 [18].

**Figure 1:**
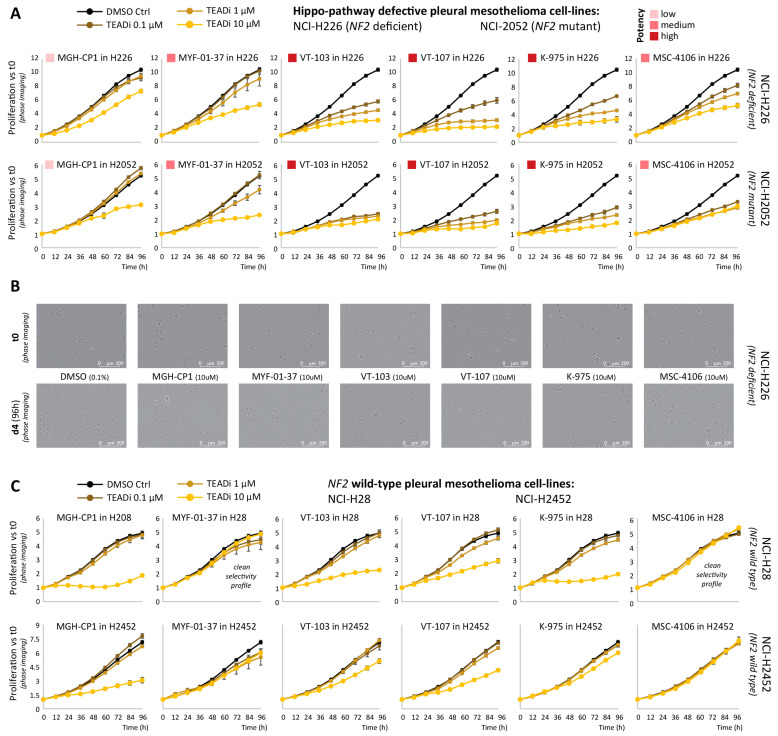
Incucyte live-cell proliferation assays. (**A**) The anti-proliferative effects of the indicated TEAD-palmitoylation inhibitors are shown for NF2 defective NCI-226 (*NF2*-deficient) and NCI-2052 (*NF2*-mutant) cell lines. (**B**) Representative images from NCI-226 cells are shown. (**C**) Same as (**A**), but *NF2* wild-type NCI-H28 and NCI-H2452 are shown. Error bars represent SD from four separate technical replicates. One of at least two independent biological replicates is shown in this figure. Please see Table S1 for biological replicates and original raw data.

First, to compare the potency and selectivity of the above-mentioned TEADi(s), we performed live-cell proliferation assays (Incucyte) in two *NF2* defective mesothelioma cell lines (NCI-H226 and NCI-H2052), using two *NF2* wild-type cell lines (NCI-H28 and NCI-H2452) as a compare/contrast selectivity control. Each TEAD inhibitor was tested at 0.1-μM, 1.0-μM, and 10-μM doses and proliferation was monitored by phase imaging every 12 h over the course of 4 days. As shown in Figure 1A, TEADi(s) potency varied, with VT-103, VT-107, and K-975 displaying the strongest inhibition of proliferation in the two *NF2* defective cell lines. MYF-01-37 and MSC-4106 had an intermediate potency, whereas MG-CP1 showed limited effects and only at the high, 10-μM dose. Notably, none of the TEADi(s) tested was able to induce a complete block of proliferation, with at least one population doubling observed during the 4-day time course. No cellular fragmentation nor formation of apoptotic bodies could be observed by phase imaging (Figure 1B). Finally, the 10-μM dosing of VT-103, VT-107, K-975, and MC-CP1 affected the proliferation of NF2 WT cell lines as well (mostly NCI-H28, but similar trends could be observed for NCI-H2452), whereas no effect was observed with MYF-01-37 and MSC-4106 in these cell lines, suggesting a cleaner target profile, albeit coupled with a slightly reduced potency (Figure 1C). Similar outcomes were obtained when using an orthogonal, end-point viability assessment (CellTiterGLO, Promega), performed at either 2 days (48 h) or 5 days after drug treatment (Figure S1A,B and Table S1).

**Figure 2:**
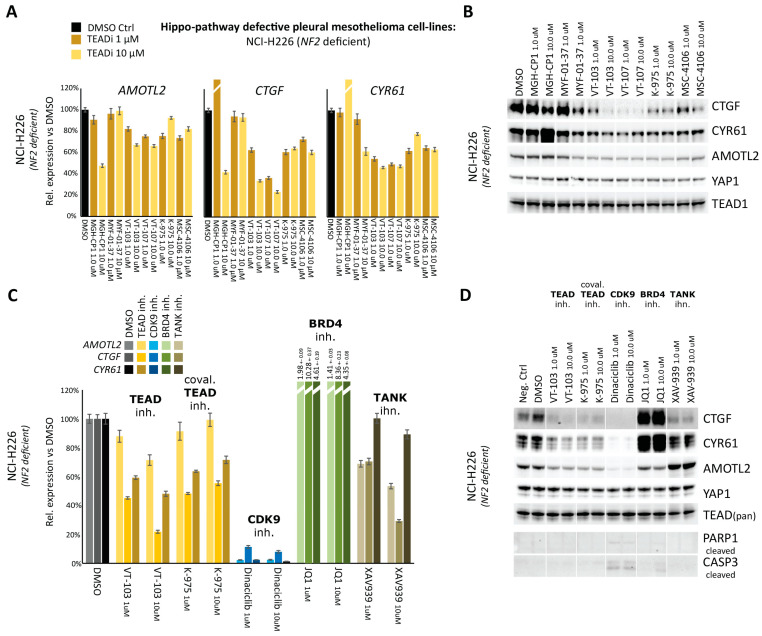
Phenotypic characterization of TEAD-palmitoylation inhibitors. (**A**) RT-QPCR: the effects of the indicated TEAD-palmitoylation inhibitors on the mRNA expression of three bona-fide YAP/TEAD target-genes (*AMOTL2*, *CTGF*, and *CYR61*) are shown for *NF2*-deficeint NCI-226 cells after a 24 h drug treatment (See Figure S2A for NCI-H2052 cells). (**B**) Western-Blot: the effects of the indicated TEAD-palmitoylation inhibitors on the protein expression of AMOTL2, CTGF, and CYR61 are shown for *NF2*-deficient NCI-226 cells after a 24 h drug treatment. Antibodies against Yap1 and Tead1 were used as loading control (See Figure S2B for NCI-H2052 cells). (**C**) RT-QPCR: the effects of the indicated small molecule inhibitors on the mRNA expression of three bona-fide YAP/TEAD target-genes (*AMOTL2*, *CTGF*, and *CYR61*) are shown for *NF2*-deficient NCI-226 cells after a 24 h drug treatment (See Figure S2C for NCI-H2052 cells). (**D**) Western-Blot: the effects of the indicated small molecule inhibitors on the protein expression of AMOTL2, CTGF, and CYR61 are shown for *NF2*-deficientNCI-226 cells after a 24 h drug treatment. Antibodies against Yap1 and Tead1 were used as loading control. Antibodies against cleaved-Parp1 and cleaved-Casp3 were used to monitor apoptosis induction (See Figure S2D for NCI-H2052 cells). RT-QPCR error bars represent SD from three separate technical replicates. One of at least two independent biological replicates is shown in this figure. Please see Table S2 for biological replicates and original raw data.

Next, we performed RT-QPCR to directly measure the effects of TEAD inhibitors in blocking the expression of three bona-fide YAP/TEAD target genes: *AMOTL2* (*Angiomotin Like 2*), *CTGF* (*Connective tissue growth factor*), and *CYR61* (*Cysteine-rich angiogenic inducer 61*). As shown in Figure 2A, the efficacy of TEADi(s) in this phenotypic assay was overall limited, and less than 50% reduction in YAP/TEAD target genes expression was observed for most drug-gene pairs. Notable exceptions to this trend were VT-103 and VT-107 that led to a stronger inhibition of *CTGF* and, to a lesser extent, *CYR61* in *NF2*-deficient NCI-H226 cells (see Figure S2A for H2052 cells), consistent with their high potency in the proliferation assays. Western Blot analyses performed after TEADi treatment strongly aligned with the RT-qPCR findings (Figure 2B and Figure S2B), suggesting the limited efficacy of TEAD-palmitoylation inhibitors might result, at least in part, from the failure to fully block the expression of YAP/TEAD oncogenic signatures.

To expand on these observations, we compared two TEADi(s) (VT-103, reversible, and K-975, covalent) with other small molecules target cofactors of YAP/TEAD transcriptional activity, such as CDK9 (Cyclin-dependent kinase 9) [32,33] and BRD4 (Bromodomain-Containing Protein 4) [34], or inhibit non-transcriptional modulator of YAP/TEAD activity, such as tankyrases [35]. As shown in Figure 2C (see Figure S2C for H2052 cells), abrogation of transcriptional elongation via CDK9 inhibition (Dinaciclib) resulted in the complete blockage of (>90%) of YAP/TEAD transcriptional output. These findings support the idea that TEADi(s) fail to robustly block YAP/TEAD dependent transcription is intrinsic to this drug-modality, and it is not an indirect consequence of long half-life of *AMOTL2*, *CTGF*, and *CYR61* mRNAs. Tankyrases inhibition (XAV933), leading to YAP sequestration via stabilization of angiomotins, phenocopied the partial effects of TEAD inhibition, whereas blockage of BRD4 (JQ1) unexpectedly led to an increase in the expression of YAP/TEAD target genes, at least at the time points and concentration tested. Western blot analyses (Figure 2D and Figure S2D) confirmed the RT-qPCR findings, with AMOTL2, CTGF, and CYR61 proteins being essentially undetectable following CDK9 inhibition. Importantly, complete blockage of YAP/TEAD transcriptional output following CDK9 inhibition correlated with apoptosis priming, as assessed by cleaved-Parp1 and cleaved-Caspase-3 blots, whereas no apoptosis induction was observed following TEADi(s) treatment by itself.

**Figure 3:**
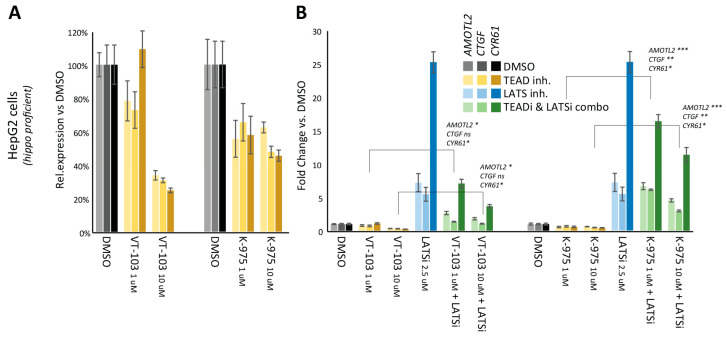
LATS1/2 inhibition overcomes the effects of TEAD-palmitoylation inhibitors. (**A**) RT-QPCR: the effects of the indicated TEAD-palmitoylation inhibitors (yellow bars) on the mRNA expression of 3 bona-fide YAP/TEAD target genes (*AMOTL2*, *CTGF*, and *CYR61*) are shown for Hippo-proficient HepG2 cells after a 4 h drug treatment. (**B**) RT-QPCR: the effects of the indicated small molecule inhibitors on the mRNA expression of three bona-fide YAP/TEAD target genes (*AMOTL2*, *CTGF*, and *CYR61*) are shown for Hippo-proficient HepG2 cells after a 4 h drug treatment. Treatments are color-coded as follows: TEADi in yellow, LATSi in blue, and combo-treatments in green. RT-QPCR error bars represent SD from three separate technical replicates. One of at least two independent biological replicates is shown in this figure. T-statistics were calculated from two independent biological replicates. Please see Table S3 for biological replicates, original raw data and T-statistics calculations. * *p* < 0.2, ** *p* < 0.05 and *** *p* < 0.01.

Finally, because of the central role of LATS1/2 kinases in restricting YAP/TEAD activity in the Hippo pathway-proficient cell lines, we sought to combine TEADi(s) (VT-103, reversible, and K-975, covalent) with a selective LATS1/2 kinase inhibitor [36], to test if the increase in YAP activity resulting from LATS1/2 inhibition was per se sufficient to overcome the effects of TEADi on YAP/TEAD dependent transcription. In the Hippo pathway-proficient cell line HepG2, TEAD inhibition led to a partial reduction in *AMOTL2*, *CTGF,* and *CYR61* mRNA expression, as expected and consistent with the partial efficacy we observed in Hippo-defective mesothelioma cell lines (Figure 3A).

Conversely, LATS1/2 kinase inhibition led to a strong transactivation of YAP/TEAD target-genes, as expected (Figure 3B, blue bars). In co-treatments, LATS1/2 inhibition was overall dominant, and whereas the addition of a TEADi(s) reduced the magnitude of the transactivation observed, (Figure 3B, green bars), the net effect of combo-treatments was still a robust induction of YAP/TEAD target genes comparted to the DMSO control, even in the presence of significantly high-doses (10 μM) of TEADi(s). The same was true for both the reversible (VT-103) and covalent (K-975) TEADi, further supporting the idea small molecule inhibitors of TEAD-palmitoylation cannot completely block TEAD dependent transcription.

To summarize our finding so far, we generated a coherent single-agent dataset informing on the potency and selectivity of several TEAD-palmitoylation inhibitors. These analyses highlighted an overall limited efficacy for this drug-modality, suggesting TEAD palmitoylation might serve as a “regulatory dimmer” for TEAD function rather than an actual on-off switch. This observation prompted us to explore the behavior of TEAD inhibitors in drug-combination settings.

### 2.2. High-Throughput Mapping of TEAD-Palmitoylation Inhibitors Drug-Combination Landscape

To systematically map the drug-combination landscape of TEADi in Hippo-deficient mesothelioma, we performed a high-throughput combinatorial drug-screening between the TEADi VT-103 and an entire collection of about 2800, mechanistically annotated, oncology-focused drugs (MIPE 6.0 library, Mechanism Interrogation PlatEs version 6.0) in NCI-H226 (*NF2*-deficient) and NCI-2052 (*NF2*-mutant) cell lines. This library exploits redundancy by including multiple inhibitors against well-explored oncogenic targets while simultaneously encompassing mechanistic diversity, targeting more than 800 distinct mechanisms of action (MoA) [37,38].

**Figure 4:**
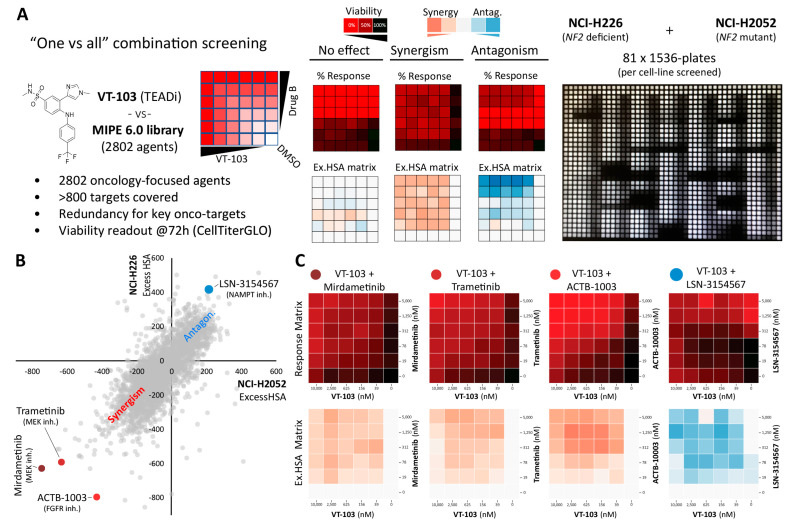
TEADi high-throughput combinatorial drug-screening. (**A**) Schematic layout of the drug-versus-all screen for VT-103 versus the entire MIPE 6.0 library. Left: each drug pair was tested in a 6 × 6 matrix block, reflecting five doses plus DMSO control for each individual drug. Middle: exemplar outcomes (no effect, synergy, or antagonism) are displayed as % response or ExcessHSA heat-maps. Right: an image of an actual 1536-well plate containing multiple 6 × 6 blocks. (**B**) ExcessHSA scatter plot highlighting the consistent outcomes between the NCI-226 (*NF2*-deficent) and NCI-2052 (*NF2*-mutant) cells. Selected HITs are highlighted in the plot and displayed in more detail in Figure 4C. Please see Table S4 for the complete screening outcomes. (**C**) % response or ExcessHSA heat-maps for the selected HITs highlighted in Figure 4B are shown.

Briefly, each of the 2802 TEADi drug-pair was assessed in a quantitative, 6 × 6, dose-response format, reflecting five doses plus DMSO control for each individual drug, generating twenty-five combination data points plus ten single-agent ones for each combination tested. For the primary screening, end-point viability (CellTiterGLO) was assessed at three days (72 h) post drug-combo treatment and the “Excess over the Highest Single Agent” (ExcessHSA) metric was used to quantitatively determine VT-103 synergism/antagonism outcomes (Figure 4A) [39]. As shown in Figure 4B, the VT-103 drug-combination landscape was highly consistent across the two Hippo-deficient cell lines tested (Pearson’s correlation 0.74), enabling us to generate an aggregate synergy/antagonism metric (AVRG ExcessHSA) for all the follow-up analyses. Based on the above, the three most synergistic HITs included two MEK (Mitogen-activated protein kinase kinase) inhibitors (Mirdametinb was the top synergistic hit and Trametinib was #3), together with the FGFR (Fibroblast growth factor receptor) inhibitor ACTB-1003 (synergistic hit #2) (Figure 4C). Several conserved drug antagonism interactions were also observed, and the antagonism between VT-103 and the NAMPT (Nicotinamide phosphoribosyltransferase) inhibitor LSN-3154567 (HIT #6 from the antagonism perspective) is highlighted on the left side of Figure 4C. Importantly, we noticed several other synergistic HITs (Table S4) belonged to well-defined target-classes, suggesting the existence of discrete oncogenic pathways that could be targeted to increase the efficacy of TEAD inhibitors.

To expand on these observations, we exploited MIPE 6.0 mechanistic redundancy to unbiasedly identify all the significant TEADi “target-level”, druggable co-dependencies. Briefly, we ranked the “VT-103 vs. MIPE 6.0” screening outcomes based on the aggregate synergism/antagonism score (AVRG ExcessHSA) and used this pre-ranked drug-universe to perform a drug-target set enrichment analysis (DTSEA) against a custom collection of “drug-target signatures” representing any MIPE 6.0 target/MoA that is covered by at least three drugs (*n* = 309). This analysis enabled us to identify fifteen target-level, TEAD co-dependencies, defined as druggable targets whose inhibition consistently synergized with TEADi VT-103 (FDR < 10%, Table S5). The three most enriched targets in the synergy-space were glucocorticoid receptors (GR) (#24 agonists in MIPE 6.0), MEK1/2 kinases (#17 inhibitors in MIPE 6.0), and mTOR kinases (mammalian target of Rapamycin, #26 inhibitors in MIPE 6.0).

**Figure 5:**
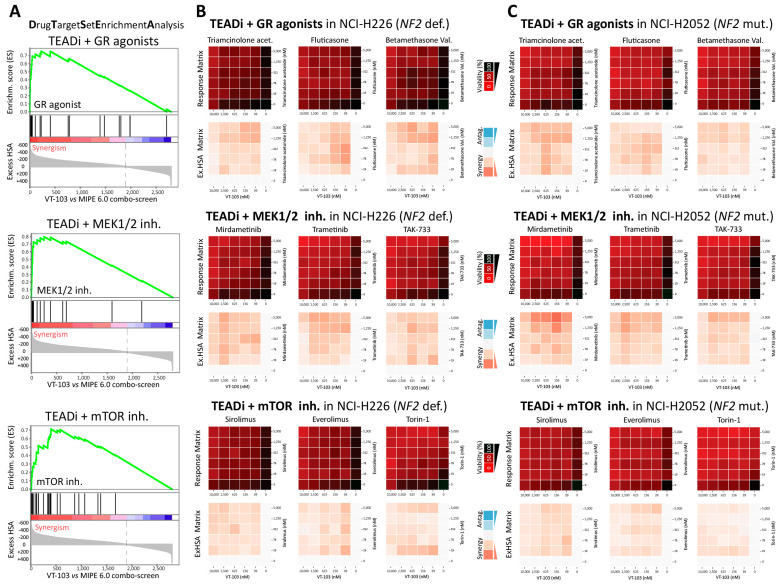
Drug Target Set Enrichment Analysis (DTSEA). (**A**) Representative enrichment plots are shown for the following synergistic drug-targets: glucocorticoid receptors (GR) (#24 agonists in MIPE 6.0), MEK1/2 kinases (#17 inhibitors in MIPE 6.0), and mTORC1/2 complexes (#26 inhibitors in MIPE 6.0). Individual drugs within each target-class are highlighted as black lines with respect to the pre-ranked “VT-103 vs. MIPE 6.0” screening outcomes. (**B**) % response or ExcessHSA heat-maps for the selected target-level co-dependencies are displayed for NCI-H226 (NF2-deficient) cells. Three representative drugs for each drug-target class are shown. (**C**) Same as (**B**), but NCI-H2052 (NF2-mutant) cells are shown.

As shown in Figure 5A, drugs within the above-mentioned target-classes showed very consistent behavior across the entire screenings, with most of the drugs within each class strongly clustering toward the synergism space. Response and excess-HSA matrices for three representative drugs within each of the top three target-class are shown in Figure 5B,C, for NCH-H226 and NCI-H2052 cells, respectively. Notably, most of these drug-pairs showed strong synergistic inhibition of mesothelioma cell proliferation at low nM doses, whereas the corresponding single-agent activities were limited, even at μM doses, suggesting a pattern of drug-to-drug interaction akin to genetic synthetic lethality. The target-level synergy list included other well-established oncogenic kinases, such as PI3Ks (Phosphoinositide 3-kinases), JAKs (Janus kinases), and Aurora kinases, together with epigenetic/transcriptional modulator such as the bromodomain, extra-terminal domain (BET) protein BRD4, and the mediator kinase CDK8 (Cyclin-dependent kinase 8) (Table S5). Finally, our analysis also identified a discrete set of targets (#5) whose inhibition antagonized the proliferative defects induced by TEAD inhibition (Figure S3). These included DHFR and NAMPT inhibitor, together with small molecule modulators of anti-apoptotic BCL2 (B-cell lymphoma 2) family members.

The latter finding was somewhat unexpected because, at least in NSCLC, YAP/TEAD transcription factors have been shown to promote resistance to RAF- and MEK-targeted therapies via transcriptional upregulation of anti-apoptotic BCL2L1 (BCL2-like 1 or BCL-xL). We thus decided to systematically test if the TEADi synergisms we observed in Hippo-defective mesothelioma were resulting from apoptosis induction. To this end, we performed a targeted drug-combination assessment in 10 × 10 format and acquired parallel readouts to measure both proliferation inhibition (CellTiterGLO at 48 h) and apoptosis induction (Casp3/7GLO at 24 h). This secondary screen profiled all the significant “target-level” HITs from the primary “one vs. all” runs and also included a compare/contrast assessment between reversible (VT-103) and the covalent (K-975) TEAD inhibitors. We performed these assays in the *NF-2* mutant cell line NCI-H2052 and in a *LATS1* deficient cell line, MSTO-211H, to expand the general relevance of our findings [40].

**Figure 6:**
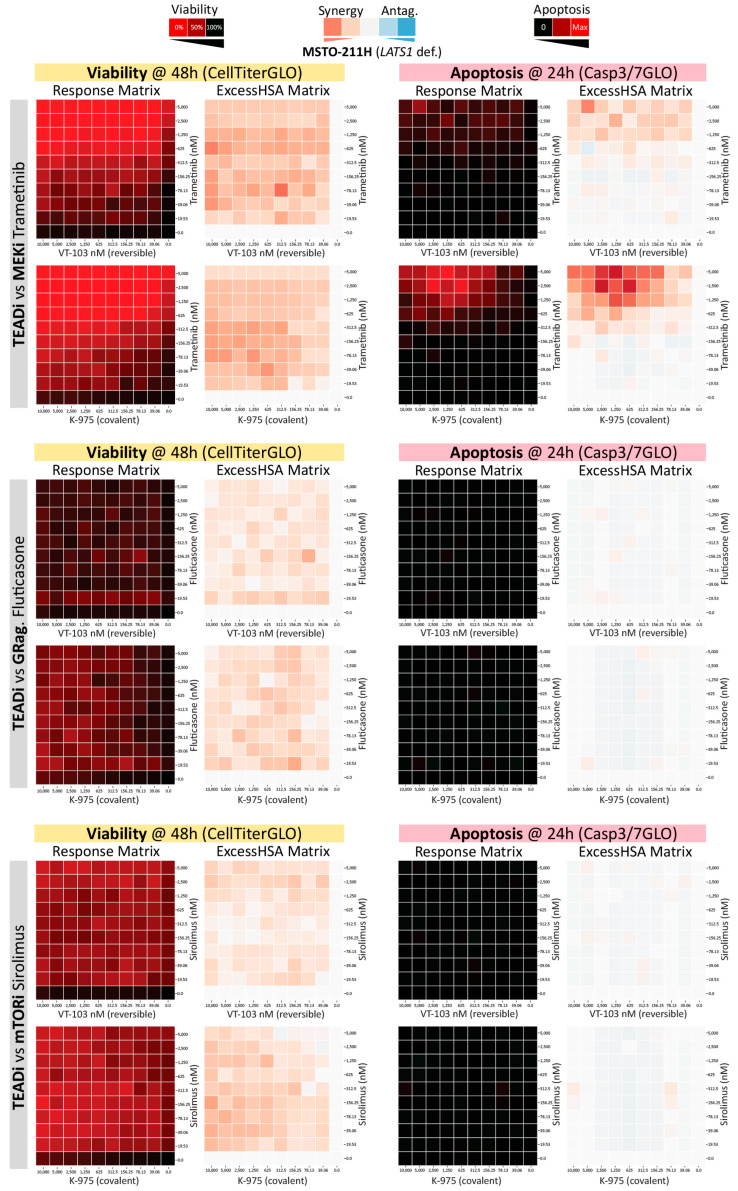
Secondary, 10 × 10 drug-combination screenings to inform on proliferation-inhibition and apoptosis induction. % response and ExcessHSA heat-maps are shown for MSTO-211H (NF2-mutant) cells. Both reversible (VT-103) and covalent (K-975) TEAD inhibitors were combined with the indicated targeted agents and proliferation inhibition (CellTiterGLO 48 h) or apoptosis induction (Casp3/7GLO 24 h) were measured in parallel.

As shown in Figure 6 (MSTO-211H, please see Figure S4 for NCI-H2052 cells), we confirmed robust synergistic effects on proliferation across all the tested drug pairs. However, the observed TEADi synergisms were largely independent of apoptosis induction, suggesting a proliferation block and/or non-apoptotic forms of cell death are the major contributors to the observed drug-interactions. This was true for most of the targeted agents tested, and no significant differences were observed between reversible and covalent TEAD inhibitors (Figure S5). One noticeable exception to these trends was the combination of TEADi and MEK1/2i (Trametinib) in MSTO-211H cells. However, induction of apoptosis occurred at doses significantly higher than those required to synergistically block proliferation, suggesting even when present, apoptosis is likely a secondary consequence, and not the major driver, of the observed drug-to-drug synergisms.

Altogether, we generated a coherent drug-combination dataset focused on TEAD-palmitoylation inhibitors in malignant pleural mesothelioma. Our comprehensive profiling enabled the unbiased identification of several druggable co-dependencies of TEAD-palmitoylation inhibitors. Whereas a detailed mechanistic understanding of all the identified drug-to-drug interactions goes beyond the scope of this work, our aggregate findings highlight the strength of this combinatorial platform to enable systematic discovery of well-defined oncogenic signaling pathways that could be explored to develop drug-combination strategies centered around TEAD inhibition. This data should support preclinical and early clinical efforts toward targeting Hippo pathway deficient mesothelioma and, more broadly, other cancer types dependent on the aberrant activation of YAP/TAZ/TEAD oncogenic transcriptional programs.

## 3. Discussion

Aberrant activation of TEAD-family transcription factors (TEADs) is a central determinant of oncogenic transformation in cancers characterized by dysregulation of the Hippo signaling pathway. Genetic inactivation of core genes within this tumor-suppressor kinase cascade (for example, about 35% to 40% of malignant pleural mesotheliomas cases carry an inactivating mutations at the neurofibromatosis 2, *NF2*, locus) or genetic amplification of YAP/TAZ transcriptional regulator (for example, more than 15% of cervical squamous cell carcinoma are driven by YAP/TAZ amplification) converge on TEAD transcription factors to establish an oncogenic transcriptional program that promotes uncontrolled cell proliferation [6,10]. This convergence highlights TEAD’s centrality and establishes TEAD as a classic example of “non-oncogene addiction” that could be targeted therapeutically [41].

Whereas transcription factors have often been considered ‘undruggable’ by small molecules due to a high degree of structural disorder and lack of a well-defined small molecule binding pockets [42], the discovery of a lipophilic auto-palmitoylation cavity shared by all TEAD family members has provided an opportunity to develop TEAD-palmitoylation inhibitors, TEADi(s). Several efforts from both academia and pharma have been described [29,43,44] with two different TEAD-palmitoylation inhibitor, VT3989 and IK-930, currently undergoing Phase I clinical evaluation in patients with advanced/metastatic solid tumors (ClinicalTrials.gov ID NCT04665206 and NCT05228015, respectively). Whereas the results from these ongoing clinical studies will establish if interfering with TEAD-palmitoylation is sufficient to induce durable clinical benefits in the above cancer patients’ population, our analysis in cell line derived models of malignant pleural mesothelioma highlighted an overall limited efficacy for this drug-modality, likely resulting from the fact that TEAD-palmitoylation inhibitors fail to completely block TEAD dependent transcription. Our findings are consistent with previous reports on individual TEADi [17,27], and strongly suggest TEAD-palmitoylation serves as a “regulatory dimmer” for TEAD function rather than a bona-fide on-off switch. Importantly, our mechanistic understanding of these regulatory events is partial, and small molecules that bind to this pocket and activate TEAD-dependent transcription have also been reported [45].

Because of the above limitations, we decided to systematically explore the potential of TEAD inhibitors in combination settings. Our comprehensive profiling enabled the unbiased identification of several druggable co-dependencies of TEAD-palmitoylation inhibitors in malignant pleural mesothelioma. These included well-established drug-targets for oncology indications, such as glucocorticoid receptors (GR), MEK1/2, mTOR, PI3Ks, JAKs, and Aurora kinases, together with the epigenetic factor BRD4. Combination of TEADi with inhibitors of the above-mentioned targets led to consistent and robust inhibition of mesothelioma cell proliferation, often highlighting a pattern of drug-to-drug interaction akin to genetic synthetic lethality.

Whereas the functional interplay between TEADs and MAPK signaling pathway has been previously established [19,30,46,47,48], most of the TEADi “target-associations” we describe in this study here are novel and uncharacterized. Hence, we hope this dataset could serve as a rational foundation to support preclinical and early clinical efforts toward the development of targeted, drug-combination regimens centered around TEAD inhibition is Hippo pathway dysregulated cancers.

In this context, it is important to note none of the TEADi drug-pairs we identified led to a robust apoptotic response, suggesting the observed synergisms are mostly cytostatic in nature. From a translational standpoint, this constitutes a limitation, because the development of therapies effectively eliminating and killing cancer cells has been a mainstay and goal of clinical oncology for over three decades [49].

The lack of a strong apoptotic priming in these settings could be a consequence of the partial blockage of YAP/TEAD transcriptional output, and it is plausible to imagine a more complete blockage of TEAD oncogenic function could better prime cells for apoptosis, as we have indeed observed following complete TEAD blockade via CDK9 inhibition. Fortunately, due to the ongoing and increasing interest in this relatively untapped drug-target, this field is extremely dynamic and, as scientists continue to explore alternative modalities of TEAD inhibition, the hope that second-generation TEAD inhibitors might help address some of the limitations highlighted in this study is high. For example, Novartis recently described a series of biaryl-derivatives as direct YAP/TAZ-TEAD protein-protein interaction (PPI) inhibitors [50], and the clinical candidate derived from this series, IAG933 (ClinicalTrials.gov ID NCT04857372) elicited complete tumor regression in a xenograft model of LATS1/2 deficient mesothelioma (MSTO-211H) [51]. Even more recently, Genentech reported a pan-TEAD inhibitor, GNE-7883, allosterically blocking the interactions between YAP/TAZ and all human TEAD paralogs [52].

Further studies aimed at comparing/contrasting these second-generation TEAD PPI inhibitors with first-generation palmitoylation blockers will be required to properly dissect the contributions of each TEADi drug-modality with respect to efficacy, potency, and more importantly translational potential. Finally, because YAP/TAZ and TEADs are discrete cancer dependencies that can be separated by lineage-specific determinants, and are not always unequivocally intertwined [30,53,54], the development of small molecule inhibitors specifically target either YAP or TEAD, possibly via proteolysis targeting chimera (PROTAC), would be a much-welcomed addition to the current therapeutic arsenal to attack these central transcriptional targets.

## 4. Methods

### 4.1. Cell Culture

Malignant pleural mesothelioma cell lines NCI-H28 (*NF2* wild-type), NCI-H2452 (*NF2* wild-type) NCI-H226 (*NF2*-deficient), NCI-H2052 (*NF2* mutant), MSTO-211H (LATS-deficient) were purchased from ATCC. All mesothelioma cell lines were grown in RPMI-1640 medium supplemented with Glutamax (Gibco), 10% FBS (Cytiva, Marlborough, MA, USA), and 1% penicillin/streptomycin (Gibco). HepG2 cells were purchased from ATCC and grown in MEM medium supplemented with Glutamax (Gibco), 10% FBS (Cytiva), and 1% penicillin/streptomycin (Gibco). All cell lines were maintained in a humidified, 5% CO_2_ incubator at 37 °C.

### 4.2. Drugs

Drugs were purchased for commercial sources or synthesized in house, as follow. Dinaciclib: Axon Medchem. SNS-032: Bio Vision. MGH-CP1: Enamine. MYF-01-37, VT-103, VT-107, K-975, XAV939: MedChem Express. Mivebresib (ABBV-075): Selleck. JQ1: Tocris. XAV939: XcessBio. MSC-4106, VT02956, LATSi (NCGC00886782): in-house. 10 mM stock solutions in DMSO were used as the starting point for all the dose-titration experiments.

### 4.3. RNA Extraction and RT-QPCR

For RNA-based assays, 5 × 10^5^ cells/well were seeded in 12 well/plates one day before treatment (seeding volume 1 mL/well). 500X stock solutions of the indicated drugs, or the corresponding volume of DMSO, were added to each well 24 h post-seeding. Drug-treated cells were incubated for the indicated times, mostly 24 h. Total RNA was extracted with the RNeasy Mini kit using a vacuum manifold (Qiagen, Hilden, Germany). 1 μg of total RNA was used for cDNA synthesis using the SuperScript^TM^ IV VILO^TM^ Master Mix (Invitrogen, Waltham, MA, USA). The cDNA template was diluted 1:20 and used to set-up RT-qPCR on a ViiA 7 Real-Time PCR System, using Taqman probes and TaqMan™ Fast Advanced Master Mix (Applied Biosystem, Waltham, MA, USA). The TaqMan assays used for this study are as follows: ACTB (Housekeeping control) probe Hs01060665_g1; AMOTL2 probe Hs01048101_m1; CTGF probe Hs00170014_m1; CYR61 probe Hs00155479_m1.

### 4.4. Western Blot

For protein-based assays, 1.0 × 10^6^ cells/well were seeded in 6-well plates one day before treatment (seeding volume 2 mL/well). 1000X stock solutions of the indicated drugs, or the corresponding volume of DMSO, were added to each well 24 h post-seeding. Drug-treated cells were incubated for the indicated times, mostly 24 h. Total proteins were extracted with RIPA buffer (Pierce, Appleton, WI, USA), supplemented with protease-inhibitors and phosphatase-inhibitors cocktails (Sigma, San Francisco, CA, USA). 30 μg of whole cell lysates were separated on 4–12% Bis-Tris gels and transferred to 0.45 μm nitrocellulose membranes (Invitrogen). Protein targets of interest were detected using the following antibodies: AMOTL2, Proteintech Cat. No. 23351-1-AP; CTGF, Cell-Signaling #86641; CYR61, Cell-Signaling #14479; YAP1, Cell-Signaling #14074; TEAD1, Cell-Signaling #12292; Pan-TEAD, Cell-Signaling #13295; Cleaved-PARP1, Cell-Signaling #5625; Cleaved-CASP3, Cell-Signaling #9579.

### 4.5. Incucyte Live-Cell Proliferation Assays

For live-cell proliferation assays, 2.5 × 10^3^ cells/well were seeded in 96 well/plates one day before treatment (seeding volume 100 μL/well). The seeding media was replaced with drug-containing media (or DMSO, 0.1% final) 24 h post-seeding (final volume 100 μL/well). Phase imaging was performed on an Incucyte SX5 system, with image acquisition every 12 h, starting 1 h post-drug administration.

### 4.6. End-Point Proliferation Assays

For end-point proliferation assays, 2.5 × 10^3^ cells/well were seeded in 96 well/plates one day before treatment (seeding volume 100 μL/well). The seeding media was replaced with drug-containing media (or DMSO, 0.1% final) 24 h post-seeding (final volume 100 μL/well). 50 μL of Cell Titer Glo reagent (Promega, Madison, WI, USA) were added to each well at the indicated time point. Luminescence readings were taken using a Viewlux imager (PerkinElmer, Waltham, MA, USA) with a 10″ exposure. Relative viability of drug-treated cells was assessed with respect to DMSO treated cells (0.1% final).

### 4.7. Quantitative High-Throughput Combination Screening (qHTCS)

Drug combination screenings were performed as previously described [37,38]. Briefly, 10 nL of compounds were acoustically dispensed into 1536-well white polystyrene tissue culture-treated plates with an Echo 550 acoustic liquid handler (Labcyte, San Jose, CA, USA). Cells were then added to compound-containing plates at a density of 500-cells/well in 5 μL of medium. A 5-point custom concentration range, with constant 1:4 dilution was used for all the MIPE 6.0 drugs in the primary 6 × 6 matrix screening against TEADi VT-103 (1:4 dilution), A 9-point custom concentration range was used for all the secondary validation runs in 10 × 10 matrix format. For primary screens, plates were incubated for 72 h at standard incubator conditions covered by a stainless steel gasketed lid to prevent evaporation. 3 μL of CellTiterGLO (Promega) were then added to each well, and plates were incubated at room temperature for 15 min with the stainless-steel lid in place. Luminescence readings were taken using a Viewlux imager (PerkinElmer) with a 2 s exposure time per plate. Secondary 10 × 10 screenings were performed as indicated above, with a 48 h timepoint for viability assessments (CellTiterGLO) and a 24 h timepoint for apoptosis (Casp3/7GLO, Promega) assessments.

### 4.8. Drug-Target Set Enrichment Analysis (DTSEA)

To systematically map the drug-combination landscape of TEADi in Hippo deficient mesothelioma we used the Excess over the Highest Single Agent (ExcessHSA) [39] metric to quantitatively assess synergism and antagonism throughout the VT-103 vs. MIPE 6.0 drug-combination screenings. We then ranked the entire MIPE 6.0 drug-universe based on the average ExcessHSA score in NCI-H226 (*NF2*-deficient) and NCI-H2052 (*NF2*-mutant) cells. We used this ranking to run a pre-ranked Drug-Target Set Enrichment Analysis (DTSEA), against a custom collection of drug-target sets representing any MIPE 6.0 drug-target is covered by at least three small molecule inhibitors (*n*  =  308). The pre-ranked enrichment analysis was performed using the GSEA software (v4.0.3)71 with a weighted enrichment statistic [55].

## 5. Conclusions

Over recent years, the lipophilic auto-palmitoylation pocket on TEAD transcription factors has been exploited to develop TEAD-palmitoylation inhibitors for the treatment of Hippo pathway deficient cancers. Our analysis in cell line derived models of pleural mesothelioma highlighted an overall limited efficacy for this drug-modality in single-agent settings. At the same time, we identified several druggable TEAD co-dependencies involving well-defined, therapeutically relevant, oncogenic signaling pathways. This data should serve as a rational foundation to develop drug-combination strategies centered around TEAD inhibition via either palmytoilation blockage, protein-protein interaction (PPI) modulators or proteolysis targeting chimeras (PROTACs).

## Data Availability

The primary (6 × 6) and secondary (10 × 10) drug-combination screening datasets presented in this study will be available upon request from to the corresponding author. These datasets were not deposited in PubChem because PubChem assay format is limited to individual compound/substance ID per well, and does not have the capacity to accommodate drug -combination dataset. ExcessHSA values for the entire “one vs all” screenings are also reported in Table S4.

## Supplementary Materials

The following supporting information can be downloaded at: https://www.mdpi.com/article/10.3390/ph16121635/s1, Figure S1: CellTiterGLO end-point proliferation assays; Figure S2: Phenotypic characterization of TEAD-palmytoilation inhibitors in NCI-H2052 cells; Figure S3: Drug Target Set Enrichment Analysis (DTSEA); Figure S4: Secondary, 10 × 10 drug-combination screenings to inform on proliferation-inhibition and apoptosis induction, part I; Figure S5: Secondary, 10 × 10 drug-combination screenings to inform on proliferation-inhibition and apoptosis induction, part II; Table S1: TEAD inhibitors proliferation assessments; Table S2: TEAD inhibitors RT-QPCR, raw data; Table S3: TEAD inhibitors vs LATS inhibitor RT-QPCR, raw data; Table S4: VT-103 “one vs all” drug-combination screening outcomes; Table S5: Drug Target Set Enrichment Analysis (DTSEA) outcomes.

## References

[1] Yu F.X., Zhao B., Guan K.L. (2015). Hippo Pathway in Organ Size Control, Tissue Homeostasis, and Cancer. Cell.

[2] Yu F.X., Zhao B., Panupinthu N., Jewell J.L., Lian I., Wang L.H., Zhao J., Yuan H., Tumaneng K., Li H. (2012). Regulation of the Hippo-YAP pathway by G-protein-coupled receptor signaling. Cell.

[3] Zhao B., Li L., Wang L., Wang C.Y., Yu J., Guan K.L. (2012). Cell detachment activates the Hippo pathway via cytoskeleton reorganization to induce anoikis. Genes Dev..

[4] Zhao B., Wei X., Li W., Udan R.S., Yang Q., Kim J., Xie J., Ikenoue T., Yu J., Li L. (2007). Inactivation of YAP oncoprotein by the Hippo pathway is involved in cell contact inhibition and tissue growth control. Genes Dev..

[5] Zhao B., Li L., Tumaneng K., Wang C.Y., Guan K.L. (2010). A coordinated phosphorylation by Lats and CK1 regulates YAP stability through SCF(beta-TRCP). Genes Dev..

[6] Wang Y., Xu X., Maglic D., Dill M.T., Mojumdar K., Ng P.K., Jeong K.J., Tsang Y.H., Moreno D., Bhavana V.H. (2018). Comprehensive Molecular Characterization of the Hippo Signaling Pathway in Cancer. Cell Rep..

[7] Kulkarni A., Chang M.T., Vissers J.H.A., Dey A., Harvey K.F. (2020). The Hippo Pathway as a Driver of Select Human Cancers. Trends Cancer.

[8] Faraji F., Ramirez S.I., Anguiano Quiroz P.Y., Mendez-Molina A.N., Gutkind J.S. (2022). Genomic Hippo Pathway Alterations and Persistent YAP/TAZ Activation: New Hallmarks in Head and Neck Cancer. Cells.

[9] Yin F., Yu J., Zheng Y., Chen Q., Zhang N., Pan D. (2013). Spatial organization of Hippo signaling at the plasma membrane mediated by the tumor suppressor Merlin/NF2. Cell.

[10] Sekido Y., Sato T. (2023). NF2 alteration in mesothelioma. Front. Toxicol..

[11] Pobbati A.V., Han X., Hung A.W., Weiguang S., Huda N., Chen G.Y., Kang C., Chia C.S., Luo X., Hong W. (2015). Targeting the Central Pocket in Human Transcription Factor TEAD as a Potential Cancer Therapeutic Strategy. Structure.

[12] Chan P., Han X., Zheng B., DeRan M., Yu J., Jarugumilli G.K., Deng H., Pan D., Luo X., Wu X. (2016). Autopalmitoylation of TEAD proteins regulates transcriptional output of the Hippo pathway. Nat. Chem. Biol..

[13] Noland C.L., Gierke S., Schnier P.D., Murray J., Sandoval W.N., Sagolla M., Dey A., Hannoush R.N., Fairbrother W.J., Cunningham C.N. (2016). Palmitoylation of TEAD Transcription Factors Is Required for Their Stability and Function in Hippo Pathway Signaling. Structure.

[14] Kim N.G., Gumbiner B.M. (2019). Cell contact and Nf2/Merlin-dependent regulation of TEAD palmitoylation and activity. Proc. Natl. Acad. Sci. USA.

[15] Mesrouze Y., Aguilar G., Meyerhofer M., Bokhovchuk F., Zimmermann C., Fontana P., Vissieres A., Voshol H., Erdmann D., Affolter M. (2022). The role of lysine palmitoylation/myristoylation in the function of the TEAD transcription factors. Sci. Rep..

[16] Noritsugu K., Suzuki T., Dodo K., Ohgane K., Ichikawa Y., Koike K., Morita S., Umehara T., Ogawa K., Sodeoka M. (2023). Lysine long-chain fatty acylation regulates the TEAD transcription factor. Cell Rep..

[17] Holden J.K., Crawford J.J., Noland C.L., Schmidt S., Zbieg J.R., Lacap J.A., Zang R., Miller G.M., Zhang Y., Beroza P. (2020). Small Molecule Dysregulation of TEAD Lipidation Induces a Dominant-Negative Inhibition of Hippo Pathway Signaling. Cell Rep..

[18] Kaneda A., Seike T., Danjo T., Nakajima T., Otsubo N., Yamaguchi D., Tsuji Y., Hamaguchi K., Yasunaga M., Nishiya Y. (2020). The novel potent TEAD inhibitor, K-975, inhibits YAP1/TAZ-TEAD protein-protein interactions and exerts an anti-tumor effect on malignant pleural mesothelioma. Am. J. Cancer Res..

[19] Kurppa K.J., Liu Y., To C., Zhang T., Fan M., Vajdi A., Knelson E.H., Xie Y., Lim K., Cejas P. (2020). Treatment-Induced Tumor Dormancy through YAP-Mediated Transcriptional Reprogramming of the Apoptotic Pathway. Cancer Cell.

[20] Li Q., Sun Y., Jarugumilli G.K., Liu S., Dang K., Cotton J.L., Xiol J., Chan P.Y., DeRan M., Ma L. (2020). Lats1/2 Sustain Intestinal Stem Cells and Wnt Activation through TEAD-Dependent and Independent Transcription. Cell Stem Cell.

[21] Lu T., Li Y., Lu W., Spitters T., Fang X., Wang J., Cai S., Gao J., Zhou Y., Duan Z. (2021). Discovery of a subtype-selective, covalent inhibitor against palmitoylation pocket of TEAD3. Acta Pharm. Sin. B.

[22] Tang T.T., Konradi A.W., Feng Y., Peng X., Ma M., Li J., Yu F.X., Guan K.L., Post L. (2021). Small Molecule Inhibitors of TEAD Auto-palmitoylation Selectively Inhibit Proliferation and Tumor Growth of NF2-deficient Mesothelioma. Mol. Cancer Ther..

[23] Fan M., Lu W., Che J., Kwiatkowski N.P., Gao Y., Seo H.S., Ficarro S.B., Gokhale P.C., Liu Y., Geffken E.A. (2022). Covalent disruptor of YAP-TEAD association suppresses defective Hippo signaling. Elife.

[24] Gridnev A., Maity S., Misra J.R. (2022). Structure-based discovery of a novel small-molecule inhibitor of TEAD palmitoylation with anticancer activity. Front. Oncol..

[25] Hu L., Sun Y., Liu S., Erb H., Singh A., Mao J., Luo X., Wu X. (2022). Discovery of a new class of reversible TEA domain transcription factor inhibitors with a novel binding mode. Elife.

[26] Li Y., Li Y., Ning C., Yue J., Zhang C., He X., Wang Y., Liu Z. (2022). Discovering inhibitors of TEAD palmitate binding pocket through virtual screening and molecular dynamics simulation. Comput. Biol. Chem..

[27] Sun Y., Hu L., Tao Z., Jarugumilli G.K., Erb H., Singh A., Li Q., Cotton J.L., Greninger P., Egan R.K. (2022). Pharmacological blockade of TEAD-YAP reveals its therapeutic limitation in cancer cells. Nat. Commun..

[28] Lu W., Fan M., Ji W., Tse J., You I., Ficarro S.B., Tavares I., Che J., Kim A.Y., Zhu X. (2023). Structure-Based Design of Y-Shaped Covalent TEAD Inhibitors. J. Med. Chem..

[29] Pobbati A.V., Kumar R., Rubin B.P., Hong W. (2023). Therapeutic targeting of TEAD transcription factors in cancer. Trends Biochem. Sci..

[30] Barbosa I.A.M., Gopalakrishnan R., Mercan S., Mourikis T.P., Martin T., Wengert S., Sheng C., Ji F., Lopes R., Knehr J. (2023). Cancer lineage-specific regulation of YAP responsive elements revealed through large-scale functional epigenomic screens. Nat. Commun..

[31] Heinrich T., Peterson C., Schneider R., Garg S., Schwarz D., Gunera J., Seshire A., Kotzner L., Schlesiger S., Musil D. (2022). Optimization of TEAD P-Site Binding Fragment Hit into In Vivo Active Lead MSC-4106. J. Med. Chem..

[32] Galli G.G., Carrara M., Yuan W.C., Valdes-Quezada C., Gurung B., Pepe-Mooney B., Zhang T., Geeven G., Gray N.S., de Laat W. (2015). YAP Drives Growth by Controlling Transcriptional Pause Release from Dynamic Enhancers. Mol. Cell.

[33] Lu Y., Wu T., Gutman O., Lu H., Zhou Q., Henis Y.I., Luo K. (2020). Phase separation of TAZ compartmentalizes the transcription machinery to promote gene expression. Nat. Cell Biol..

[34] Zanconato F., Battilana G., Forcato M., Filippi L., Azzolin L., Manfrin A., Quaranta E., Di Biagio D., Sigismondo G., Guzzardo V. (2018). Transcriptional addiction in cancer cells is mediated by YAP/TAZ through BRD4. Nat. Med..

[35] Wang W., Li N., Li X., Tran M.K., Han X., Chen J. (2015). Tankyrase Inhibitors Target YAP by Stabilizing Angiomotin Family Proteins. Cell Rep..

[36] Ceribelli D.D.M., Hoyt S., Morris P., Tosto F.A., Thomas C.J. (2023). LATS Inhibitors and Uses. Thereof. Patent.

[37] Lin G.L., Wilson K.M., Ceribelli M., Stanton B.Z., Woo P.J., Kreimer S., Qin E.Y., Zhang X., Lennon J., Nagaraja S. (2019). Therapeutic strategies for diffuse midline glioma from high-throughput combination drug screening. Sci. Transl. Med..

[38] Mott B.T., Eastman R.T., Guha R., Sherlach K.S., Siriwardana A., Shinn P., McKnight C., Michael S., Lacerda-Queiroz N., Patel P.R. (2015). High-throughput matrix screening identifies synergistic and antagonistic antimalarial drug combinations. Sci. Rep..

[39] Foucquier J., Guedj M. (2015). Analysis of drug combinations: Current methodological landscape. Pharmacol. Res. Perspect..

[40] Miyanaga A., Masuda M., Tsuta K., Kawasaki K., Nakamura Y., Sakuma T., Asamura H., Gemma A., Yamada T. (2015). Hippo pathway gene mutations in malignant mesothelioma: Revealed by RNA and targeted exon sequencing. J. Thorac. Oncol..

[41] Luo J., Solimini N.L., Elledge S.J. (2009). Principles of cancer therapy: Oncogene and non-oncogene addiction. Cell.

[42] Henley M.J., Koehler A.N. (2021). Advances in targeting ‘undruggable’ transcription factors with small molecules. Nat. Rev. Drug Discov..

[43] Zagiel B., Melnyk P., Cotelle P. (2022). Progress with YAP/TAZ-TEAD inhibitors: A patent review (2018-present). Expert Opin. Ther. Pat..

[44] Crawford J.J., Bronner S.M., Zbieg J.R. (2018). Hippo pathway inhibition by blocking the YAP/TAZ-TEAD interface: A patent review. Expert Opin. Ther. Pat..

[45] Pobbati A.V., Mejuch T., Chakraborty S., Karatas H., Bharath S.R., Gueret S.M., Goy P.A., Hahne G., Pahl A., Sievers S. (2019). Identification of Quinolinols as Activators of TEAD-Dependent Transcription. ACS Chem. Biol..

[46] Pham T.H., Hagenbeek T.J., Lee H.J., Li J., Rose C.M., Lin E., Yu M., Martin S.E., Piskol R., Lacap J.A. (2021). Machine-Learning and Chemicogenomics Approach Defines and Predicts Cross-Talk of Hippo and MAPK Pathways. Cancer Discov..

[47] Lin L., Bivona T.G. (2016). The Hippo effector YAP regulates the response of cancer cells to MAPK pathway inhibitors. Mol. Cell Oncol..

[48] Lin L., Sabnis A.J., Chan E., Olivas V., Cade L., Pazarentzos E., Asthana S., Neel D., Yan J.J., Lu X. (2015). The Hippo effector YAP promotes resistance to RAF- and MEK-targeted cancer therapies. Nat. Genet..

[49] Carneiro B.A., El-Deiry W.S. (2020). Targeting apoptosis in cancer therapy. Nat. Rev. Clin. Oncol..

[50] Furet P., Bordas V., Le Douget M., Salem B., Mesrouze Y., Imbach-Weese P., Sellner H., Voegtle M., Soldermann N., Chapeau E. (2022). The First Class of Small Molecules Potently Disrupting the YAP-TEAD Interaction by Direct Competition. ChemMedChem.

[51] Schmelzle T., Chapeau E., Bauer D., Chene P., Faris J., Fernandez C., Furet P., Galli G., Gong J., Harlfinger S. (2023). IAG933, a selective and orally efficacious YAP1/WWTR1(TAZ)-panTEAD protein-protein interaction inhibitor with pre-clinical activity in monotherapy and combinations. Cancer Res..

[52] Hagenbeek T.J., Zbieg J.R., Hafner M., Mroue R., Lacap J.A., Sodir N.M., Noland C.L., Afghani S., Kishore A., Bhat K.P. (2023). An allosteric pan-TEAD inhibitor blocks oncogenic YAP/TAZ signaling and overcomes KRAS G12C inhibitor resistance. Nat. Cancer.

[53] Lin K.C., Moroishi T., Meng Z., Jeong H.S., Plouffe S.W., Sekido Y., Han J., Park H.W., Guan K.L. (2017). Regulation of Hippo pathway transcription factor TEAD by p38 MAPK-induced cytoplasmic translocation. Nat. Cell Biol..

[54] Lin K.C., Park H.W., Guan K.L. (2017). Regulation of the Hippo Pathway Transcription Factor TEAD. Trends Biochem. Sci..

[55] Subramanian A., Tamayo P., Mootha V.K., Mukherjee S., Ebert B.L., Gillette M.A., Paulovich A., Pomeroy S.L., Golub T.R., Lander E.S. (2005). Gene set enrichment analysis: A knowledge-based approach for interpreting genome-wide expression profiles. Proc. Natl. Acad. Sci. USA.

