# Supplementary material for: Comparative Assessment and High-Throughput Drug-Combination Profiling of TEAD-Palmitoylation Inhibitors in Hippo Pathway Deficient Mesothelioma
Source: Pharmaceuticals (Basel). 2023 Nov 21;16(12):1635. doi: 10.3390/ph16121635 (PMC10747288; doi:10.3390/ph16121635)
Supplement: Supplementary file 1 [file pharmaceuticals-16-01635-s001.zip › Supplementary Figures.pdf]

Supplementary Figures: "Comparative Assessment and High-Throughput Drug-Combination Profiling of TEAD-Palmitoylation Inhibitors in Hippo Pathway Deficient Mesothelioma".

Lale Evsen[1], Patrick J. Morris[1], Craig J. Thomas[1] and Michele Ceribelli[1,*]

[1]Division of Preclinical Innovation, National Center for Advancing Translational Sciences (NCATS), National Institutes of Health (NIH), Rockville MD 20850, USA.

*Correspondence: michele.ceribelli@nih.gov

Supplementary Figures

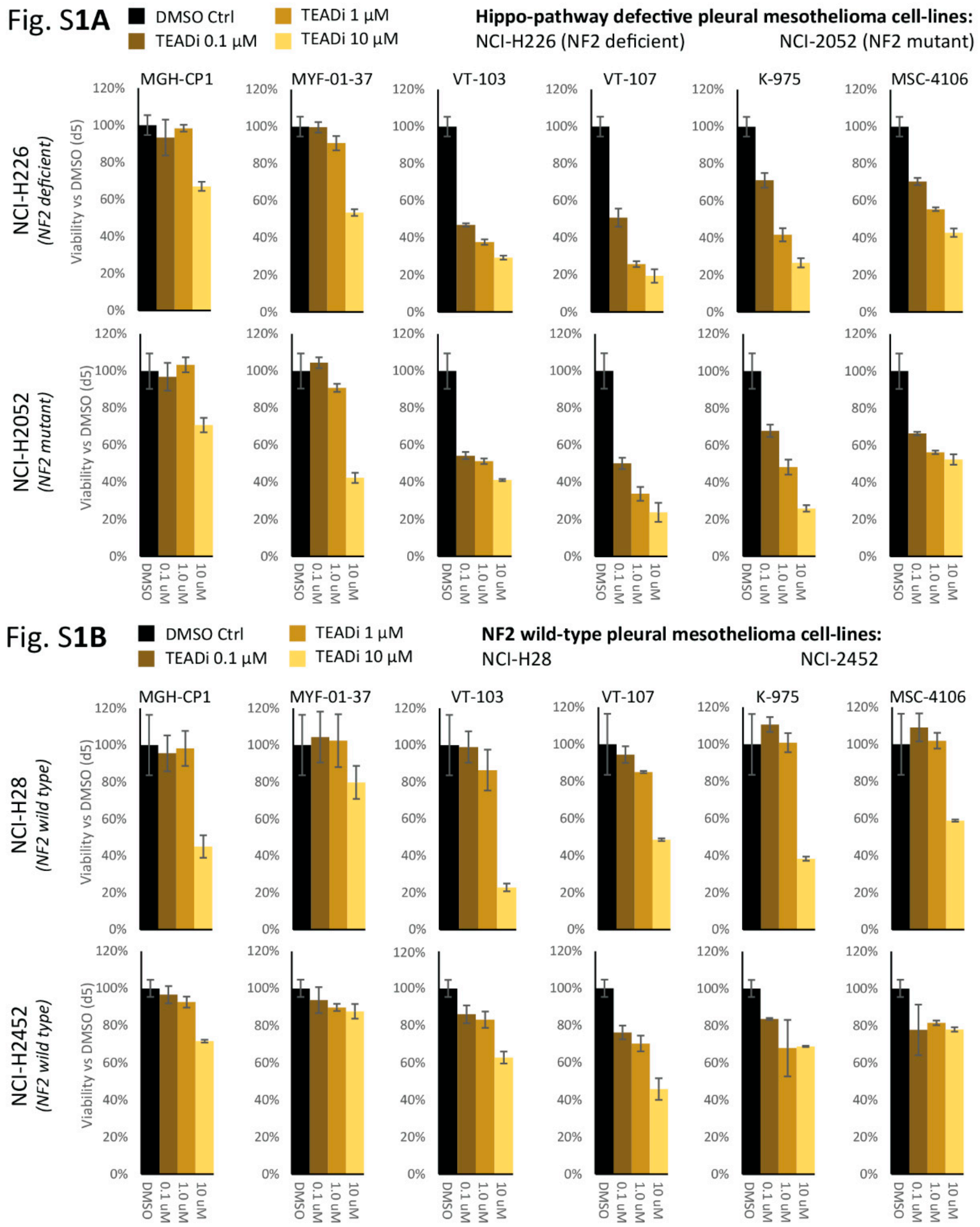

Figure S1. *CellTiterGLO end-point proliferation assays. (**A**) The anti-proliferative effects of the indicated TEAD-palmytoilation inhibitors are shown for NF2 defective NCI-226 (NF2 deficient) and NCI-2052 (NF2 mutant) cell-lines. (**B**) Same as (B), but NF2 wild-type NCI-H28 and NCI-H2452 are shown. Error bars represent SD from 4 separate technical replicates. One of at least two independent biological replicates is shown in this figure. Please see Table S1 for biological replicates and original row data.*

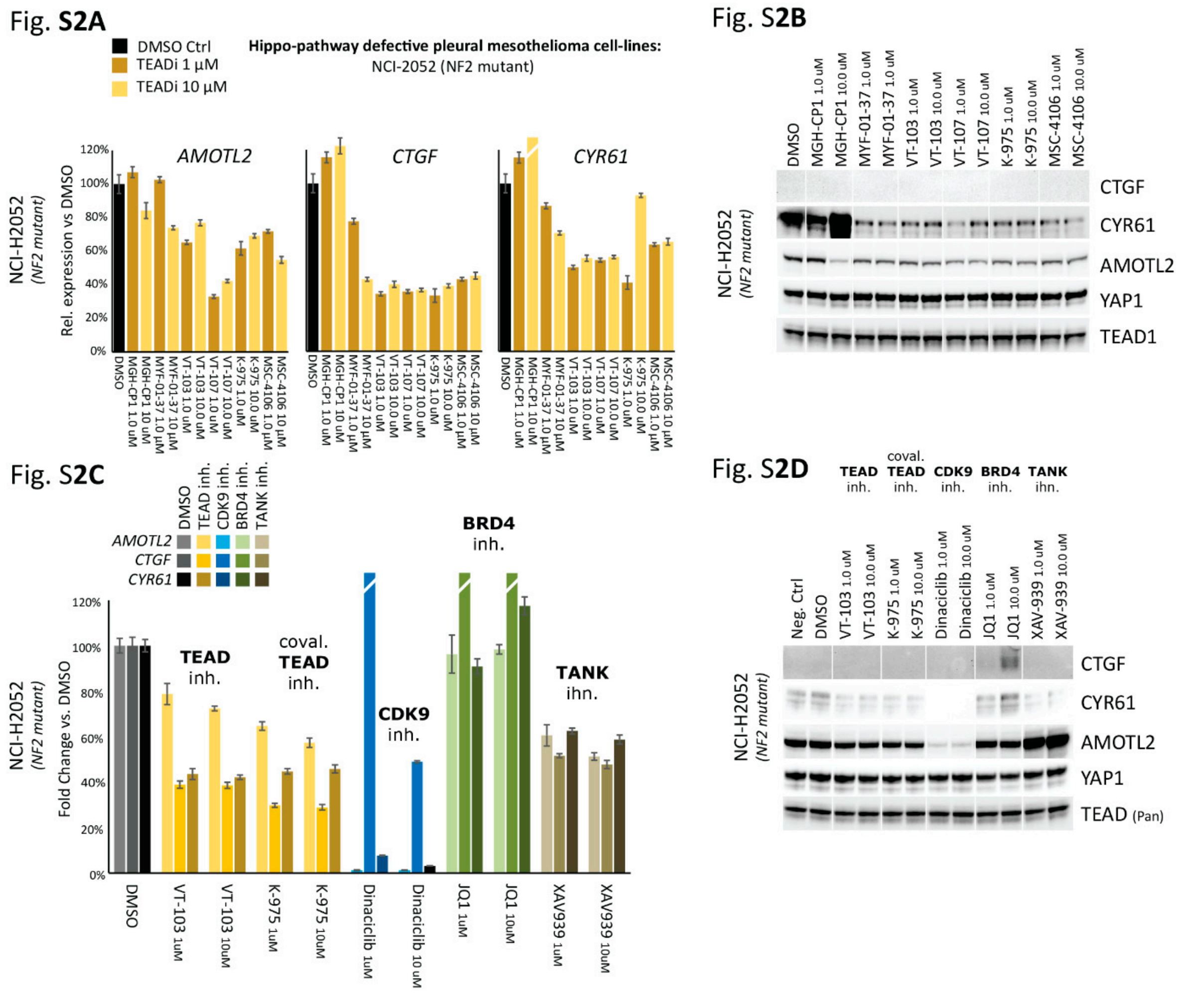

Figure S2. *Phenotypic characterization of TEAD-palmytoilation inhibitors in NCI-H2052 cells. (**A**) RT-QPCR: the effects of the indicated TEAD-palmytoilation inhibitors on the mRNA expression of three bona-fide YAP/TEAD target-genes (AMOTL2, CTGF and CYR61) are shown for NF2-mutant NCI-2052 cells after a 24h drug-treatment. (**B**) Western-Blot: the effects of the indicated TEAD-palmytoilation inhibitors on the protein expression of Amotl2, Ctgf and Cyr61 are shown for NF2-mutant NCI-2052 cells after a 24h drug-treatment. Antibodies against Yap1 and Tead1 were used as loading control. (**C**) RT-QPCR: the effects of the indicated small-molecule inhibitors on the mRNA expression of three bona-fide YAP/TEAD target-genes (AMOTL2, CTGF and CYR61) are shown for NF2-mutant NCI-H2052 cells after a 24h drug-treatment. (**D**) Western-Blot: the effects of the indicated small-molecule inhibitors on the protein expression of Amotl2, Ctgf and Cyr61 are shown for NF2-mutant NCI-2052 cells after a 24h drug-treatment. Antibodies against Yap1 and Tead1 were used as loading control. RT-QPCR error bars represent SD from 3 separate technical replicates. One of at least two independent biological replicates is shown in this figure. Please see Table S2 for biological replicates and original row data.*

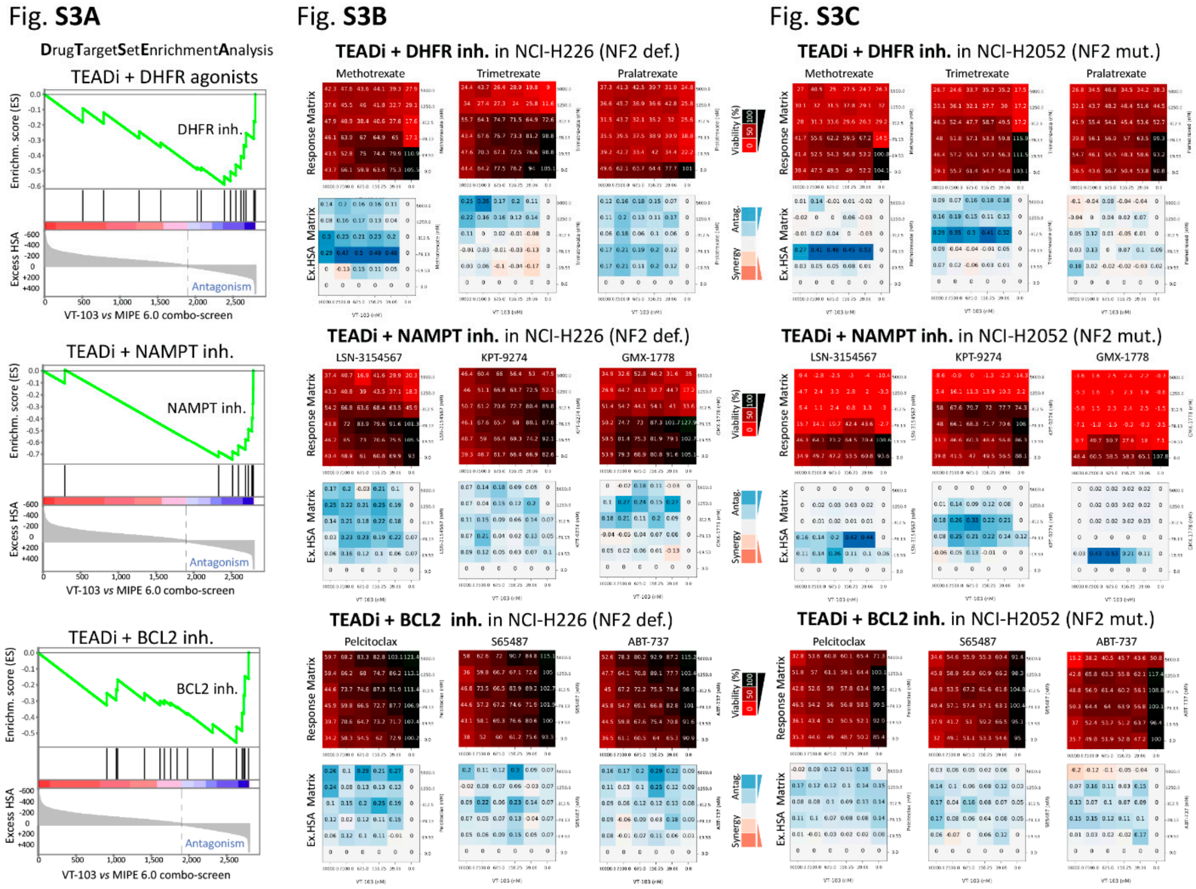

Figure S3: *Drug Target Set Enrichment Analysis (DTSEA). (**A**) Representative enrichment plots are shown for the following antagonistic drug-targets: DHFR (#14 inhibitors in MIPE 6.0), NAMPT (#10 inhibitors in MIPE 6.0) and BCL2 family members (#15 inhibitors in MIPE 6.0). Individual drugs within each target-class are highlighted as black lines with respect to the pre-ranked "VT-103 vs MIPE 6.0" screening outcomes. (**B**) % response or ExcessHSA heat-maps for the selected target-level anti-dependencies are displayed for NCI-H226 (NF2-deficient) cells. 3 representative drugs for each drug-target class are shown. (**C**) % Same as (B), but NCI-H2052 (NF2-mutant) are shown.*

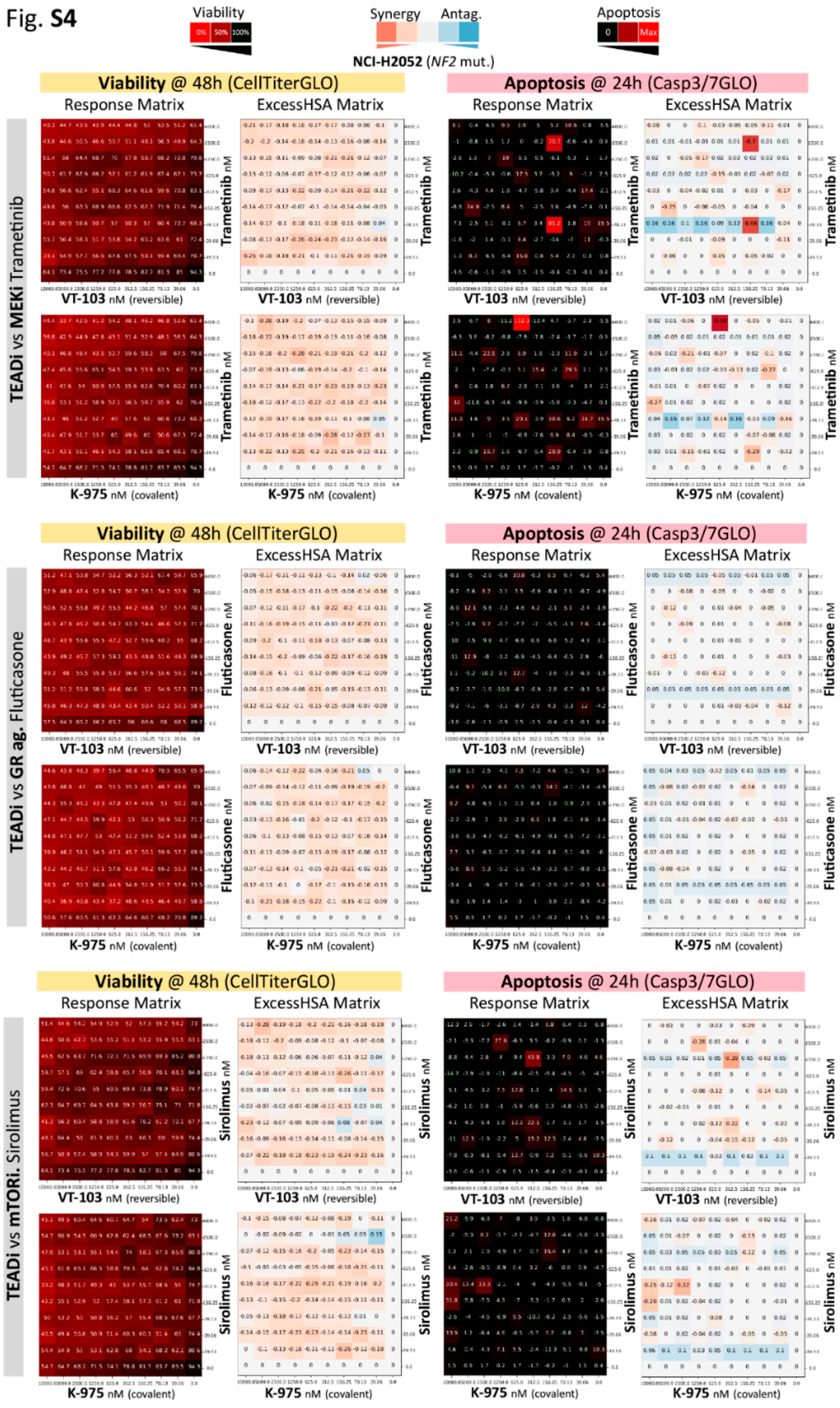

***Figure S4**: Secondary, 10 x 10 drug-combination screenings to inform on proliferation-inhibition and apoptosis induction, part I. (**A**) % response or ExcessHSA heat-maps for NCI-H2052 (NF2-mutant) cells. Both reversible (VT-103) and covalent (K-975) TEAD inhibitors were combined with the indicated targeted agents and proliferation inhibition (CellTiterGLO 48h) or apoptosis induction (Casp3/7GLO 24h) were measured in parallel.*

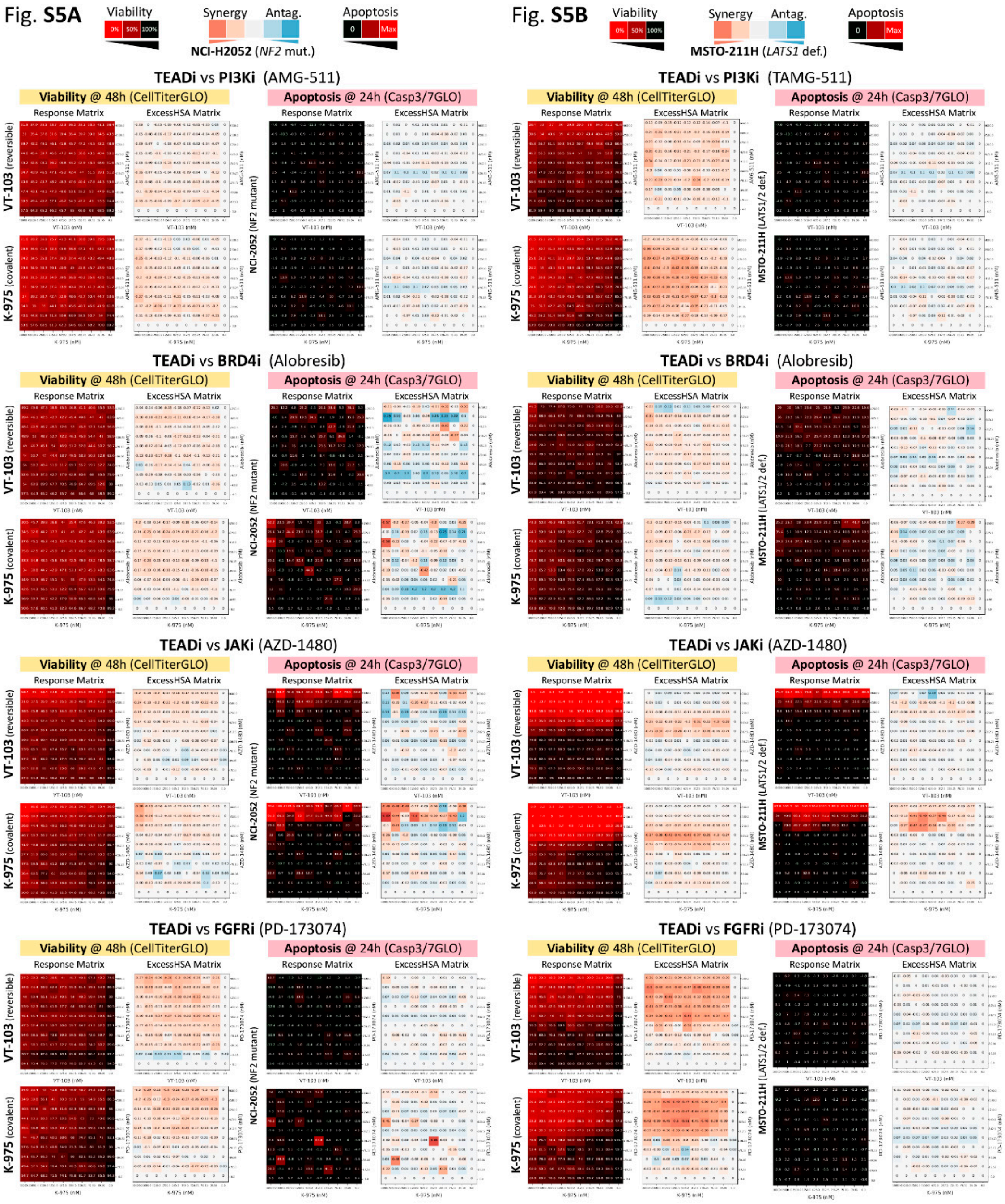

Figure S5: Secondary, 10 x 10 drug-combination screenings to inform on proliferation-inhibition and apoptosis induction, part I. (A) % response or ExcessHSA heat-maps for NCI-H2052 (NF2-mutant) cells. Both reversible (VT-103) and covalent (K-975) TEAD inhibitors were combined with the indicated targeted agents and proliferation inhibition (CellTiterGLO 48h) or apoptosis induction (Casp3/7GLO 24h) were measured in parallel. (B) Same as (A), but MSTO-211H (LATS1/2 deficient) cells are shown.
